# Using Blood Informative Transcripts in Geographical Genomics: Impact of Lifestyle on Gene Expression in Fijians

**DOI:** 10.3389/fgene.2012.00243

**Published:** 2012-11-09

**Authors:** Artika Praveeta Nath, Dalia Arafat, Greg Gibson

**Affiliations:** ^1^Center for Integrative Genomics, School of Biology, Georgia Institute of TechnologyAtlanta, GA, USA; ^2^College of Medicine, Nursing and Health Sciences, Fiji National UniversitySuva, Fiji

**Keywords:** epidemiological transition, gene expression profiling, body mass index, TLR signaling, axes of variation

## Abstract

In previous geographical genomics studies of the impact of lifestyle on gene expression inferred from microarray analysis of peripheral blood samples, we described the complex influences of culture, ethnicity, and gender in Morocco, and of pregnancy in Brisbane. Here we describe the use of nanofluidic Fluidigm quantitative RT-PCR arrays targeted at a set of 96 transcripts that are broadly informative of the major axes of immune gene expression, to explore the population structure of transcription in Fiji. As in Morocco, major differences are seen between the peripheral blood transcriptomes of rural villagers and residents of the capital city, Suva. The effect is much greater in Indian villages than in Melanesian highlanders and appears to be similar with respect to the nature of at least two axes of variation. Gender differences are much smaller than ethnicity or lifestyle effects. Body mass index is shown to associate with one of the axes as it does in Atlanta and Brisbane, establishing a link between the epidemiological transition of human metabolic disease, and gene expression profiles.

## Introduction

Globally, a major shift in public health burden known as the epidemiological transition has seen a shift from infectious disease to a Western profile of chronic metabolic diseases in most developing countries over the past three decades (Jamison and Mosley, 1991; Murray and Lopez, 1997). Globalization and urbanization have for example had a major influence on nutrition, eating habits, and lifestyle in the South Pacific (Hughes and Marks, 2009; Siefkenm et al., 2012). There have been significant changes in food production, processing, storage, and distribution, possibly also contributing to the epidemic of metabolic disease. In Fiji there has been a radical dietary shift from consumption of traditional food such as root crops, vegetables, and seafood, to western diets that are high in refined sugars and fats (Thaman, 1988; Lako, 2001; Mavoa and McCabe, 2008), and this is reflected in a 62% increase in fat intake since 1963 (Ulijaszek, 2009). In addition, physical activity has decreased and it is estimated that as few as 3.3% of people engage in regular strenuous activity (Schultz et al., 2007). Obesity and overweight in adults rose from 23 to 32% and 10 to 24% respectively between 1993 and 2004, increasing in both ethnicities and genders, and particularly in the urban populations (Saito, 1995; Schultz et al., 2007). Concomitantly, diabetes prevalence in adults 40 years and above has risen to 41% (Brian et al., 2010), and a Fiji National Food and Nutrition Center report (Schultz et al., 2007) has also showed that hypertension rates almost doubled from 10% in 1993 to 17% in 2004, again in both ethnicities and genders.

We have begun to investigate this transition at the molecular level by profiling variation in gene expression within and between population groups where two or more lifestyles and ethnicities are present in the same country (Idaghdour et al., 2008). Substantial heritability for gene expression is observed in whole blood (Powell et al., 2012), lymphoblast cell lines (Monks et al., 2004; Cheung et al., 2005), and tissue biopsies (Myers et al., 2007; Emilsson et al., 2008; Schadt et al., 2008), but it is also clear that environmental and cultural factors are just as important influences on individual profiles (Gibson, 2008). Studies have documented influences on immune profiles of such diverse environmental agents as exposure to pollution (Peretz et al., 2007), chemicals (Fry et al., 2007; Dakeshita et al., 2009), viral, and bacterial infection (Fink et al., 2007), pregnancy (Mason et al., 2010), and even the stress of taking exams (Kawai et al., 2007). Geographical genomic analysis of gene expression (Idaghdour et al., 2010) combines genome-wide expression profiling (GEP) with the mapping resolution of genome-wide association studies (GWAS; Gilad et al., 2009; Skelly et al., 2009; Kim and Gibson, 2010) where including geographic factors as a covariate makes possible the investigation of quantitative variation at the molecular level in human populations.

Our first study to directly contrast ethnic and environmental influences on the regulation of gene expression was through sampling peripheral blood leukocytes in genetically similar nomadic, rural, and dense urban Moroccan Amazigh populations (Idaghdour et al., 2008). More than one-third of the transcriptome was differently expressed in the three different regions suggesting an effect of environmental geography on gene expression. Several genes which are part of the immune response network varied in their mRNA abundance (Idaghdour et al., 2008). We followed up by including people of Arab decent in order to test whether geography or population structure affects expression levels through expression SNPs (eSNPs; Idaghdour et al., 2010). Again, over a third of all genes were differently expressed between rural and urban settings, but differences between Arabs and Berbers were only seen in the rural villages, not in the city of Agadir. Furthermore, there was no evidence for genotype-by-environment interactions at the level of *cis*-regulation of transcript abundance (Idaghdour et al., 2010). Here we evaluate how general this phenomenon is by surveying gene expression in another developing country, Fiji, where the local population is undergoing rapid transition as people are moving into larger cities and towns.

We utilize nanoscale quantitative RT-PCR (Spurgeon et al., 2008) for cost-effective targeted gene expression profiling, in place of expensive microarray analyses. Analyses of multiple large peripheral blood transcription studies of healthy adults in Morocco (Idaghdour et al., 2010), Brisbane (Mason et al., 2010), Atlanta (Preininger et al., submitted), and elsewhere (Berry et al., 2010; Inouye et al., 2010; Powell et al., 2012), allowed us to identify nine common axes of variation that collectively account for at least one-half of all blood transcriptional variance (Preininger et al., submitted). Just the first seven were known at the time the experiments reported here were performed, and each of these is represented by approximately 10 PCR primer pairs that amplify representative Blood Informative Transcripts (BIT). The axes themselves appear to reflect gene activity related to specific immune functions, from B and T cell signaling to metabolism, inflammation, and anti-viral response (Preininger et al., submitted). Profiling these representative BIT thus gives an impression of the overall transcriptional state of each person’s blood and immune system. Only specific axes differentiate lifestyle groups in Morocco or disease states in diverse studies, and we were interested to ask whether the same is true of Fijians.

## Materials and Methods

### Samples

**Figure 1:**
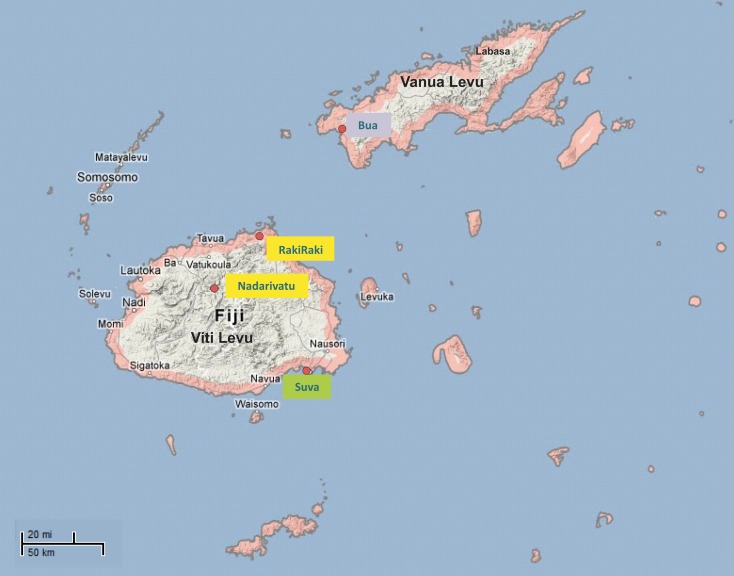
**Map of Fiji islands showing the sampling locations**. A total of 179 samples were collected from four rural Melanesian Fijian villages in two Western regions of the main island of Fiji (Viti Levu):Nadarivatu (villages of Naga and Koro) and Rakiraki (Nasavu and Nauria) highlighted in yellow, from rural Indian settlements in Northern regions of the second large island of Fiji (Vanua Levu): Bua region (Vatubogi and Luvuluvu) in blue, and from Melanesian and Indian Fijians living in the capital city of Fiji, Suva from which all of the urban samples were obtained, indicated in green.

The Fiji Islands lie in the Melanesian region of the South Pacific Ocean (18.1667′South, 178.4500′East: Figure 1). They comprise approximately 300 islands, the two largest of which are Viti Levu and Vanua Levu, where 87% of the population of nearly 860,000 reside. The two main ethnic groups are native Melanesian Fijians (57%) and Indian Fijians (38%) who mostly descend from settlers who arrived under the indenture system from southern and northern parts of India around 130 years ago (Lal, 1986). It has been estimated that 52% of the total population live in urban settings, and this proportion is projected to increase to 53.5% by 2030 (Fiji Bureau of Statistics; http://www.statsfiji.gov.fj). The remainder live in rural villages around the coastal plains, in the mountainous central regions, or on small islands. We have not included genotypic profiling in this study, but note that here is no reason to expect genetic divergence between urban and rural populations of either ethnicity, since the former are drawn essentially at random from the large pool of diversity present in Melanesians or the Indian settlers.

The study was approved by the institutional review boards of the Georgia Institute of Technology and the Fijian Ministry of Health (Fiji National Research Ethics and Review Committee). Under informed consent, approximately 10 ml peripheral blood samples were collected into LeukoLOCK filters, which size fractionate red blood cells and platelets, and retain leukocytes that are then immediately preserved in RNAlater solution (Feezor et al., 2004).The samples were obtained from 179 healthy adults from remote rural villages and from the capital city of Suva, with an approximately equal representation of both ethnicities and of men and women. All samples were collected and processed in the same manner as far as possible. The Rural Fijian and Indian samples were collected over the course of 5 days in September, 2010 and 2 days in April 2011 respectively. The urban samples were collected over 3 months from July to September, 2010. All the samples were collected in the day time between 10 a.m. and 4 p.m. They were shipped to Atlanta for RNA extraction at which point an unexpected RNA degradation issue was encountered, that required statistical adjustments during the analysis as described below.

An initial Illumina-HT12 whole transcriptome profiling experiment was performed with 41 samples of remote Melanesian villagers and urban residents. Subsequently, after removal of samples with very poor quality RNA, 124 samples were retained for Fluidigm qRT-PCR profiling.

Seventy-three samples were obtained from the rural Fijian villages of Naga, Koro, Nasavu, and Nauria which are are located in the highland district on the north side of Viti Levu Island. Naga and Koro, in the Nadarivatu region, are respectively 92 and 100 km from Tavua township and have total populations of 150 and 143 adults and children. Nasavu and Nauria are 65 and 80 km from the township of Rakiraki and have populations of 98 and 150 respectively. All four villages are mainly self sustaining with home grown fruits, vegetables, and root crops. The men are farmers who plant root crops and “kava” (*Piper methysticum*) which is the traditional Fijian drink grown by these villagers. The main source of water is tanks and springs, and Koro, Nasavu, and Nauria lack electricity with a few houses in each village having diesel generators for night use.

Fifty-two samples were obtained from rural Indians residents of the LuvuLuvu and Vatubogi settlements in the interior Bua district of Vanua Levu, approximately 130 km from the township of Labasa. They have total populations of 71 and 67 people respectively. The villagers are farmers and rely on home grown vegetables, lentils, and rice as their daily food. They also keep livestock such as chicken and goats as a source of income. These Indian villagers used coconut oil exclusively for cooking, whereas the Melanesian villagers and urban residents involved in this study have come to rely on processed, imported vegetable oil for cooking. The villagers used spring water and their source of night time electricity is diesel generators. There is no ethnic mixture between the Indian and Fijian rural localities. All the men from the two rural populations have been living in their respective localities since birth. The women typically had moved to the stated localities after marriage from other rural villages.

Fifty-four urban samples were a mixture of Melanesian (*n* = 28) and Indian (*n* = 26) Fijians residing in the capital city of Suva. All men and the majority of the women work in industries in the main city. The urban population generally consumes processed foods and purchase their fruit and vegetables from city markets.

### RNA extraction, RNA quantification and RNA quality analysis

**Figure A1:**
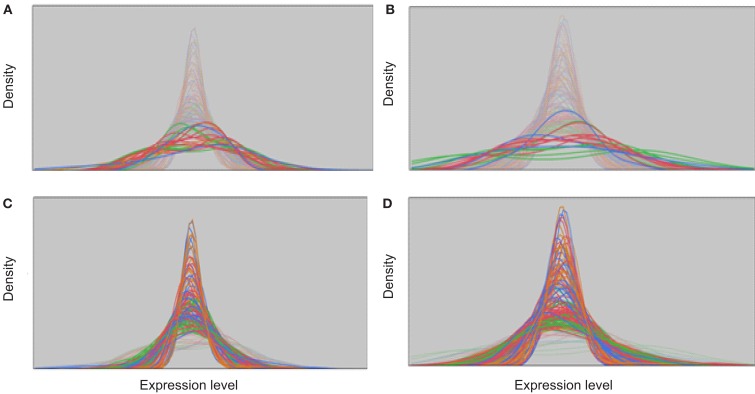
**Distribution plots of sample by RIN quality**. Distribution plots of Fluidigm Experiments I (**A,C**) and II (**B,D**) highlighting the samples that were excluded (**A,B**) or included (**C,D**) for downstream analysis on the basis of RIN quality. The *X* axis represents the range of transcript abundance and the *Y* axis represents the density for each profile.

RNA from the leukocytes was extracted using the LeukoLOCK™ Total RNA Isolation system (Ambion, now Applied Biosystems/LifeTechnologies, Carlsbad CA, USA). RNA quantity was checked using a GE NanoVue Spectrophotometer. The quality of the total RNA was determined using an RNA 6000 Nano LabChip kit in conjunction with an Agilent 2100 Bioanalyzer which contrasts the ratios of the mRNA and the 18S and 28S ribosomal RNA peaks as a measure of RNA degradation or DNA contamination. Samples that had RIN (RNA Integrity Number) greater than three were retained for potential expression profiling, though many of these were subsequently removed. Since repeated measures of RIN are often off by as much as two “points,” we classified the samples into RIN categories – BAD samples (RIN < 3 or aberrant overall profile, see Figure A1 in Appendix), POOR (3 < RIN < 6), OK (6 < RIN < 8), or GOOD (RIN > 8) – to adjust for RNA quality, as this avoids over-fitting. An additional filter based on overall distribution of Ct values led to removal of all of the very low RIN samples and a handful of the other samples that may have other issues not indicated by the Bioanalyzer analysis. This also allowed us to include some samples that would normally be discarded, while still adjusting for RIN effects by linear modeling. All analyses were also performed only on the higher quality samples.

### Gene expression profiling

Whole transcriptome profiling was performed using Illumina-HT12 bead arrays (San Diego, CA, USA) at Expression Analysis (Durham, NC, USA) using RNA extracted from the LeukoLock filters according to manufacturer’s recommendations. Raw probe-level data, namely average bead intensity for each probe from the Illumina Bead Studio, was log2 transformed, and 14,343 probes that we routinely find expressed in peripheral blood (Preininger et al., submitted) were retained for analysis, with expression of other transcripts deemed statistically indistinguishable from background.

**Figure A2:**
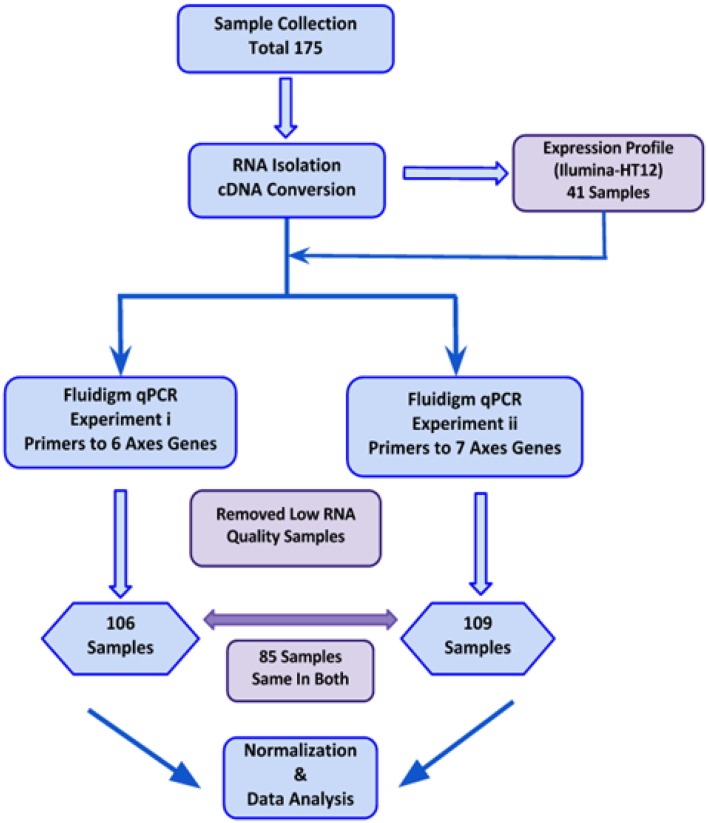
**Flow diagram of sampling strategy**.

Quantitative RT-PCR was performed using Fluidigm 96 × 96 nanofluidic arrays (Peretz et al., 2007), targeting 96 genes that we had selected to represent the seven major axes of gene expression variation in peripheral blood (Preininger et al., submitted), as well as 24 genes representing inflammatory signaling and pattern recognition receptors. Two overlapping sets of transcripts (Table S1 in Supplementary Material) were profiled in two experiments, each using two Fluidigm plates, performed 4 months apart, so as to generate partially independent representations of the axes of variation. All 41 samples were also included in the RT-PCR analyses. For the first experiment, 106 samples derived from two parallel Fluidigm arrays were analyzed after removing outlier profiles, while 109 samples were included in the analysis of the second experiment, with 86 samples included in both analyses (see Flowchart Figure A2 in Appendix). Briefly, nanoscale qRT-PCR is performed in three steps: purified total RNA is converted to single stranded cDNA using polyT priming, then all of the targeted genes are pre-amplified in a single 14-cycle PCR reaction for each sample by combining 100 ng cDNA with the pooled primers and TaqMan Pre-Amp Mastermix (Fluidigm BioMark™) following conditions outlined in the manufacturer’s protocol, and finally 9,216 parallel qRT-PCR reactions are performed for each primer pair on each sample on a 96 × 96 array. Primers were synthesized by Invitrogen, and we used the EvaGreen detection assay on a Biomark I machine following standard Fluidigm protocols.

### Statistical analysis of transcript abundance

All statistical data analysis was carried out using SAS JMP Genomics (Cary, NC; Idaghdour et al., 2010), using the standard gene expression workflow on mean-centered standardized gene expression measures after fitting a categorical measure of RNA quality to each probe. The array data was first normalized using median centering across samples. In order to ensure that RNA quality does not influence the results, RIN was categorized into three levels as stipulated above, and these were regressed against the median-centered Ct values. The residuals were converted to standardized *z*-scores and these were once more mean-centered. The resultant 96-transcript profiles are no longer affected by RIN. The positive impact of similar supervised normalization strategies are described in (Mason et al., 2010; Qin et al., 2012).

For the Illumina arrays, the standard normalized gene expression values are proportional to transcript abundance, whereas the Fluidigm values derive from Ct values and are inversely proportional to abundance (since more strongly expressed transcripts appear at an earlier cycle). Variance component analysis was performed on the first five principal components to evaluate the contributions of gender, lifestyle, and ethnicity on transcript abundance. Analysis of variance was performed at the probe-level, testing for influences of Lifestyle (Rural versus Urban), Ethnicity (Melanesian versus Indian), or the combined Type (Rural Melanesian, Rural Indian, Urban Melanesian, Urban Indian). Standard linear regression on age and BMI was also performed.

Axes of variation (Preininger et al., submitted) were extracted in clusters of up to 12 transcripts representing each of seven axes, by generating the first principal component of the correlation of the standardized probe abundance (or Ct count) for each axis, with imputation of these PC1 scores if any of the transcript measures were missing data in the Fluidigm experiments. The derivation of blood informative axes of variation is described in detail in (Preininger et al., submitted). Briefly, Chaussabel et al. (2008) identified 28 modules of conserved gene expression in peripheral blood across multiple disease classes, but we have observed that these modules reduce to seven common axes in each of more than a half dozen population-based healthy adult peripheral blood gene expression studies. Further manipulations have since revealed two additional axes. Each axis is enriched for specific gene ontologies such a T cell signaling, anti-viral response, or innate immunity. The axes are sufficiently highly conserved that it is possible to use the abundance of just 10 very strongly correlated transcripts as a proxy for the axis, and the first principal component of these “BIT” can then be used to profile the major axes of gene expression.

Analysis of variance was then used to contrast the distribution of the axes scores according to Lifestyle and Ethnicity, or linear regression was performed to test the association with anthropomorphic measures (height, BMI, age, gender). Raw and transformed data for all experiments can be obtained from the authors’ website as Table S1 in Supplementary Material, and experimental design files are in Table S2 in Supplementary Material. Axes comparison for BMI was done with the axes derived from transcriptome data of peripheral blood samples from our CHDWB (Center for Health Discovery and Well-Being) study in Atlanta (Preininger et al., submitted; Qin et al., 2012), which includes expression profiling of peripheral blood samples from 189 individual participants aged between 25 and 75 years, broadly representing the diversity of ethnic (Caucasian, Asian, and African American) and socioeconomic groups in the city.

## Results

### Microarray profile comparison of urban residents and rural melanesians

**Figure 2:**
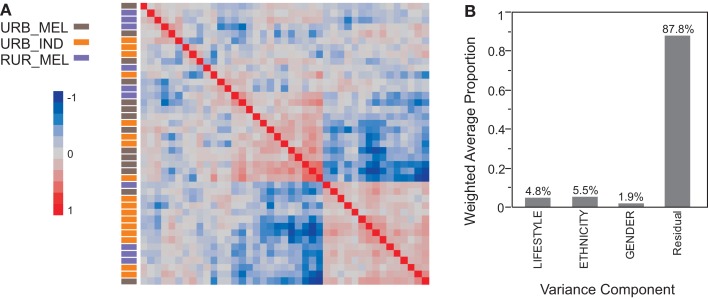
**Variance components of the illumina transcriptome**. The heat map **(A)** shows overall similarity of the expression profile across 14,343 probes by comparing each of 41 individuals to one. The brown, blue, and yellow color coding in the column to the left of the heat map indicates urban Melanesians, rural Melanesians, and urban Indians respectively. Red squares indicate strong positive correlation, blue squares indicate strong negative correlation, and lighter shades of red and blue indicates weak correlations. **(B)** Shows the weighted average of the variance captured by the first five principal components that is explained by Lifestyle, Ethnicity, and Gender.

Expression profiles generated on Ilumina-HT12 bead arrays for 41 individuals (14 urban Melanesians, 10 rural Melanesians, and 17 urban Indians) were analyzed for 14,343 probes. The correlation heat map (Figure 2A) seems to show some separation of urban Melanesians (brown samples) from rural Melanesian and urban Indians (blue and yellow samples respectively), but this differentiation is not significant since none of the first five principal components is associated with either Lifestyle or Ethnicity. Similarly, neither Age nor Gender associate with the major principal components and each factor explains less than 5% of the total variance (Figure 2B shows the contribution of the three categorical variables lifestyle, ethnicity and gender to all of the PC, weighted by the contribution of each PC to the total variance). Although the sample sizes are small, a comparison of rural villagers and urban residents in southern Morocco with similar numbers of samples did indicate differentiation of over one-quarter of the transcriptome (Idaghdour et al., 2008). This suggests that there has been relatively little modification of peripheral blood gene expression as Melanesians have adopted the urban lifestyle. Alternatively, while the highland villages are remote, there may be sufficient similarity of diet and other aspects of lifestyle to homogenize the gene expression profiles of all modern Melanesian Fijians.

### Fluidigm profile comparison of melanesians and indians from rural and urban settings

In order to extend this analysis to a larger sample including rural Indians, we next performed targeted gene expression profiling of 96 transcripts. Recent work in our lab contrasting multiple cohorts indicates that at least nine major axes of gene expression variation account for in excess of half the variance of all peripheral blood transcripts measured on microarrays. These axes are thought to reflect particular aspects of blood function from innate immune activation to inflammation and anti-viral response, and can be captured as the first principal component of just 10 transcripts that are most strongly associated with the axes (Preininger et al., submitted). We reasoned that coregulation of these BITs might also be detectable with qRT-PCR arrays, and here tested the first seven axes using two overlapping sets of BITs listed in Table S3 in Supplementary Material. Additional probes were included representing cell surface markers and intracellular inflammatory signaling pathways.

**Figure 3:**
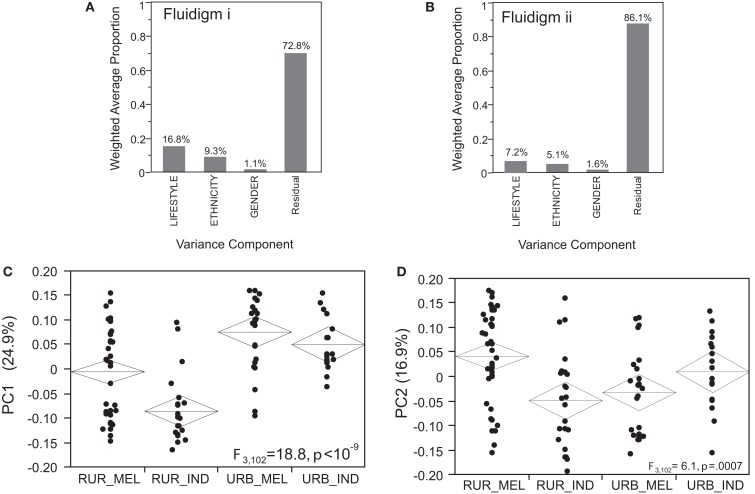
**Variance and principal component analysis of the Fluidigm experiments**. **(A,B)** The weighted average amount of variance captured by Lifestyle, Ethnicity, and Gender in each of the two Fluidigm experiments (the Gender effect is not significant by permutation). **(C,D)** Each plot shows the PC1 **(A)** or PC2 **(B)** score for each individual in Fluidigm experiment I, clustered by lifestyle and ethnicity for the four localities: rural Melanesian (RUR_MEL), rural Indians (RUR_IND), urban Melanesians (URB_MEL), and urban Indians (URB_IND). **(C)** PC1 appears to be divergent in Rural Indians (rural Indians lower, *p* < 10^−9^). **(D)** PC2 also appears to be divergent in urban Melanesians, but in experiment 2 it is only the rural Indians who have low scores, and the median urban Melanesian score here is elevated.

Before describing the Axis associations, we first present the variance components analysis of the first five principal components of gene expression measured on the Fluidigm arrays. In experiment 1, there were 106 individuals with high quality profiles, and 94 probes with signal above background in most individuals. PC1 through PC5 explain 61.7% of the variance in Ct counts, and Lifestyle (Urban versus Rural) explained 16.8% of this variation while Ethnicity (Melanesian versus Indian) just 9.3% (with non-significant Gender contributions; Figure 3A). Most of these effects are due to differentiation of rural Indians from the Melanesians and urban residents for PC1, 2, and 4 as shown in Figures 3C,D and Table 1. Qualitatively similar results were obtained for experiment 2 in which 109 individuals had high quality profiles and 90 probes were included in the analysis. In this case, PC1 through PC5 explain 60.2% of the variance in Ct counts, of which 7.2% was explained by Lifestyle and 5.1% by Ethnicity (Figure 3B). Each of these contributions is significant at *p* < 0.05, as random permutation of labels with respect to expression profiles established that no more than 3% of the variation is explained by chance at this level.

**Table 1:** **Principal components of fluidigm gene expression measures**.

Principal Component	PVE I ^1^	PVE ii ^1^	LIFE I ^2^	LIFE ii ^2^	ETHN I ^2^	ETHN ii ^2^	L × E i^2^	L × E ii ^2^
PC1	24.9	13.2	1.1 × 10^−9^	1.1 × 10^−5^	9.4 × 10^−4^	0.098	0.17	0.064
PC2	16.9	19.0	0.68	0.29	0.20	0.0040	5.2 × 10^−4^	0.0051
PC3	9.4	12.9	0.47	0.25	0.97	0.40	0.24	0.64
PC4	5.6	8.7	2.1 × 10^−4^	0.12	1.1 × 10^−7^	0.69	0.063	0.31
PC5	4.9	6.4	0.55	0.0047	0.27	0.22	0.15	0.92

*^1^Percent of overall variance in standardized Ct measures explained by the PC*.

*^2^Significance of the regression on Lifestyle, Ethnicity, or of the Interaction term, in Experiments I and II*.

**Figure 4:**
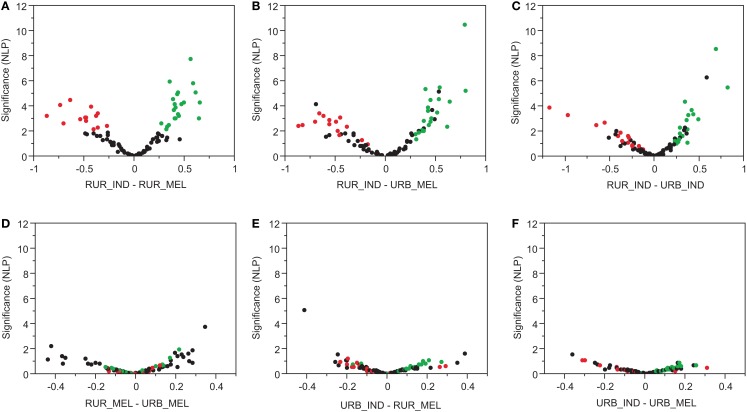
**Volcano plots of statistical significance of transcript abundance changes between the different locations and ethnicities**. For each gene, significance is indicated by negative log10 *p*-value is on the *y*-axis, and the standardized difference in log2 Ct scores on *x*-axis (positive values imply higher Ct values and hence lower expression). Pair-wise comparisons are made between the rural Fijian (RUR_FIJ), rural Indians (RUR_IND), urban Fijians (URB_FIJ), and urban Indians (URB_IND). Rural Indians are the most diverse of the groups as seen in **(A–C)**. No significant divergence was seen between the rural Fijian, urban Indians, and urban Fijians **(D–F)**.

One way ANOVA was performed to identify individual transcripts that differentiate the four combinations of lifestyle and ethnicity. The volcano plots in Figure 4 confirm that the rural Indians diverge significantly from the other three population groups. Each plot shows the fold difference in expression on the *x*-axis with significance on the *y*-axis represented as the negative logarithm of the *p*-value. Since only one gene is expected at NLP > 2 in each pair-wise comparison, we adopt this threshold as an approximate false discovery rate cut-off. Genes more highly expressed in rural Indians than rural Melanesians are colored red in Figure 4A, and those down-regulated in this comparison are in green. This contrast carries through in the comparisons of rural Indians with urban Melanesians (Figure 4B) and urban Indians (Figure 4C). Most of the down-regulated genes in the rural Indians (that is, high Ct counts and hence lower expression) are annotated to anti-viral response (*SERPING1, DDX24, OAS3, MX1, OASL, IRF1*) or inflammation/neutrophil signaling (*IKBKB, IRAK1, AKT1, ZDHHC18, NUP214*), implying a generally lower immune response. The comparisons involving rural Fijians and the urban population groups (Figures 4D–F) do not reveal any significant differences.

**Figure 5:**
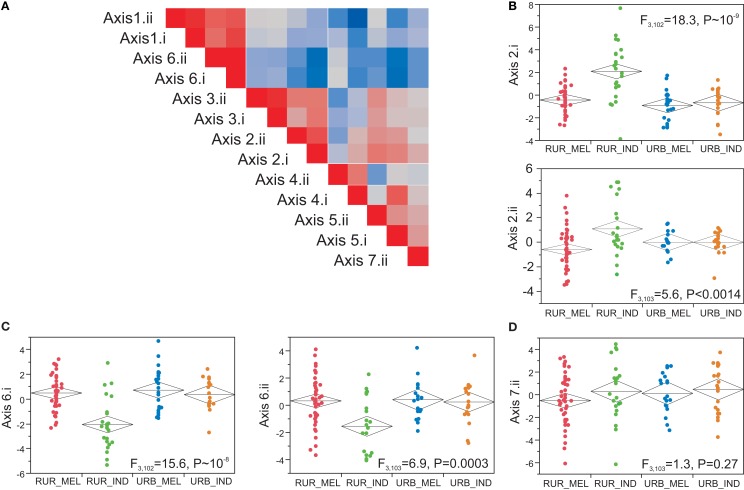
**Axes of variation**. **(A)** Correlation structure of the seven axes for Fluidigm experiments I and II. The level of correlation between axis scores is indicated by the color code (red positive, blue negative, as in Figure 2) and shows that each Axis is closely correlated with the same Axis in the other experiment. The remaining plots show the relationship between Axis scores and population sample as in Figure 3, highlighting Axes 2 **(B)** and 6 **(C)**, contrasted with a non-significant trend for divergence of Axis 7 in rural Melanesians **(D)**.

These results imply that for a targeted subset of loci that are thought to capture major axes of blood and immune function, there is clear differentiation of the rural Indians from the other individuals. To explore whether this is restricted to particular Axes, we generated PC1 for each of the seven axes in the two experiments that utilized partially overlapping probe sets. The correlation between scores for the 120 individuals in common in the two experiments are uniformly high as shown in Figure 5A and the last column of Table 2, indicating that the Axes scores replicate, and hence do capture common Axes of coregulation. Axes 1 and 6 are positively correlated, as are Axes 4 and 5 to some extent, as previously observed in other datasets, and here we also observe some similary with Axis 2. Axis 7 is enriched for anti-viral responses and is typically distinct, though it is mildly correlated with Axis 5 here (it was not represented in the experiment 1 array). To confirm that these correspond to Axes identified using Illumina microarrays, we also generated the scores for the 41 individuals included on both platforms, and consistently observed a negative correlation (results not shown), as expected since lower Ct counts correspond to higher gene expression and higher bead fluorescence.

**Table 2:** **Blood informative transcript axes**.

Axis	Experiment I			Experiment II			Correspondence^4^	
	*N* genes^1^	PC1^2^	*P*_LifeEthn_^3^	*N* genes^1^	PC1^2^	*P*_LifeEthn_^3^	Adj RSq	*P*_corr_
Axis 1	10	36.0	0.12	10	38.5	0.18	0.27	1.6 × 10^−7^
Axis 2	9	41.5	2.0 × 10^−6^	10	33.6	0.014	0.46	1.1 × 10^−12^
Axis 3	10	27.4*	0.0012	12	32.4	0.028	0.32	1.0 × 10^−8^
Axis 4	9	42.7	0.012	13	38.8	0.0019	0.16	1.1 × 10^−4^
Axis 5	11	37.1	0.0028	12	35.8	0.0039	0.11	2.9 × 10^−3^
Axis 6	10	37.1	6.6 × 10^−6^	9	39.5	0.0010	0.78	2.6 × 10^−29^
Axis 7	10	48.1	0.31	N/A			

*^1^The number of transcripts contributing to the Axis in each experiment*.

*^2^The percent of variants for the contributing transcripts that is explained by PC1 in all samples*.

*^3^The *p*-value from an ANOVA of the effect of lifestyle and ethnicity with 85 individuals measured in both experiments (the four-way contrast is of Rural Melanesian or Indian, Urban Melanesian or Indian)*.

*^4^The correspondence between Axis scores for all 85 individuals measures in both experiments, along with the significance of the regression. Note that Axis 5 shows reduced correlation because new probes were designed to distinguish it from Axis 4*.

### Association of gene expression with lifestyle and body mass index

**Figure 6:**
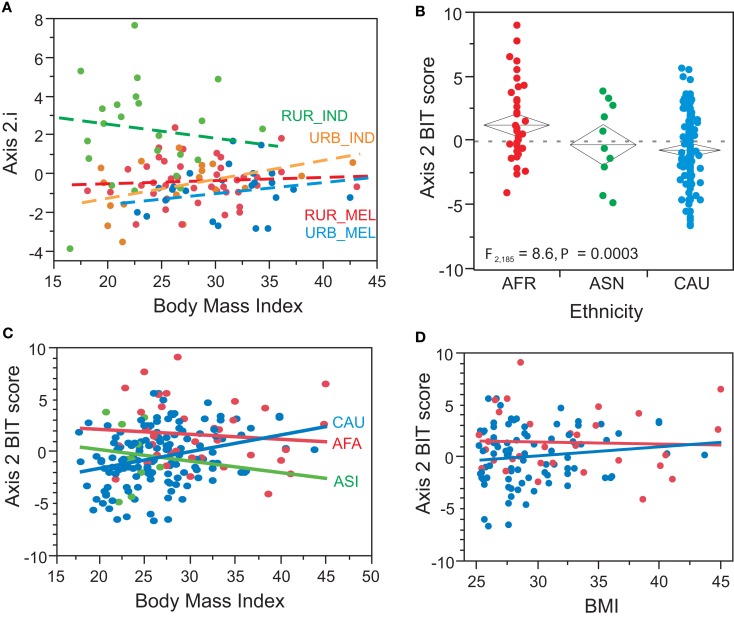
**BMI and axis 2**. Although there is an overall significant correlation between BMI and Axis 2 (*p* = 0.03), it appears to be driven largely by the low BMI in most of the rural Indians **(A)**, as the correlation is not observed in each population considered alone. In our Atlanta Center for Health Discovery and Well-being study (Gibson, 2008), African Americans have significantly elevated Axis 2 scores relative to Asians and Caucasians **(B)**, but the significant regression of BMI on Axis 2 is only seen for Caucasians **(C)**. Considering just the high BMI samples **(D)** the correlation is not observed (similarly for low BMI, not shown), suggests a transition to high Axis 2 as BMI moves into the overweight category. Note the inversion between Axis scores for Fluidigm and Illumina data due to the expression profiling measures (hybridization intensity is directly proportional to transcript abundance, whereas Ct is inversely proportional to it.

Next we asked how the Axes correlate with Lifestyle, Ethnicity, and BMI. In both Fluidigm experiments, significant divergence between rural Indians and the other three groups was seen for Axes 2, and 6, while rural Melanesians show a non-significant trend toward lower expression of Axis 7 (Figure 5). Axes 1, 4, and 5 showed much weaker influences of Lifestyle that did not replicate in both experiments (Table 2) these influences can also be attributed to covariance between these axis scores and the more significant axes. In multiple large Illumina peripheral blood gene expression profiling experiments conducted in Atlanta (Preininger et al., submitted), Brisbane (Mason et al., 2010), and Finland (Inouye et al., 2010), Axis 2 also associates with BMI. Overall, this is also true in Fiji (Figure 6A), but not when broken down by population group. Since rural Indians tend to have lower BMI than rural Melanesians, and in turn urban residents of both ethnicities, the Axis 2 association with Lifestyle may be in part due to the effect of obesity on this axis of gene expression. A similar phenomenon is observed in our Atlanta study, where there is very strong association between Axis 2 and BMI in Caucasians, but no relationship in African Americans (Figure 6C). This may reflect a true differentiation between ethnic groups, or be an artefact of the distribution of BMI scores. Splitting the Atlanta dataset into two halves, individuals considered normal weight, with a BMI less than 25, and overweight or obese individuals with BMI greater than 25, favors the latter interpretation since there is no association with BMI in either half of the data (Figure 6D). Most of the African Americans in this study have BMI greater than 25, explaining their high Axis 2 score (note that the Axes are inverted between microarrays and qRT-PCR arrays due to the nature of the expression measures). The Asians in the sample are almost all low BMI and also do not show a trend of association between Axis 2 and BMI. This may suggest a transition in Axis 2 expression as individuals move into the overweight class, and implies that the effect is more likely environmental than having a direct genetic basis. However, the divergence in rural Indians cannot be solely explained by their difference in body mass since jointly fitting BMI and location to the Indian sample absorbs the BMI effect while only slightly reducing the significance of the urban – rural comparison.

## Discussion

We have shown that Fluidigm nanoscale quantitative RT-PCR arrays can be used to probe the geographical genomics of lifestyle transitions. These arrays facilitate parallel profiling of the abundance of 96 transcripts in 96 samples. By appropriately targeting genes that are representative of variation throughout the genome, it is possible to obtain a profile of each person relatively inexpensively (currently of the order of just $20). Based on recent analyses of several large microarray datasets, we chose two sets of approximately 70 PCR amplicons that represent seven major axes of variation in the form of BIT, and document how these BIT repeatedly capture differences among population groups on the islands of Fiji.

The correlation between measures of gene expression using the targeted Fluidigm arrays and whole transcriptome microarrays in this study was somewhat less than that observed in pilot analyses of our Atlanta cohort. Three reasons are likely to have contributed: the small sample size of just 41 individuals leads to imprecise estimates with both technologies; the Fiji data was affected by relatively low RNA quality (RIN < 7) for half of the samples (though qualitatively similar results were obtained when just the high quality samples were considered; all expression measures were conditioned on RNA quality statistically); and the BIT probes have not been optimized for capturing the same information as the bead arrays, so some may perform less well or capture different splice forms.

In fact, the correspondence of alternate probes for the same gene on microarrays is often as divergent as the difference between the less strongly correlated Fluidigm and Illumina probes reported here (unpublished observations). For this reason, we repeated the analyses with a second set of probes that resulted from refinement of the definition of the Axes after completing the first experiment, and found both the results to be in agreement with each other.

The major biological finding of this study is that there is a highly significant impact of lifestyle and ethnicity on gene expression in the peripheral blood of Fijians. Specifically, rural Indian villagers on the island of Vanua Levu are markedly distinct from their urban counterparts in Suva. The rural highland Melanesians only showed modest differentiation from their urban counterparts, who are not distinguishable from the urban Indians with regards their gene expression. These results recapitulate our findings in southern Morocco (Idaghdour et al., 2008, 2010), where remote rural Berber villagers tend to have a quite different profile from those of Arab villagers and both Arab and Berber urban residents. Since we do not have genotype data on the study participants, we cannot rule out the possibility that genetic divergence contributes to the urban-rural comparisons. Comparisons of the two rural Indian villages did not show any transcriptional divergence between them, and though one of the Melanesian villages differed significantly for PC1 (result not shown), this could have either an environmental or a genetic basis. Relatedness is certainly high within villages, but marriages tend to be between villages so it is highly unlikely that there is sufficient genetic divergence to explain the gene expression patterns.

Another possible source of differentiation is regulatory polymorphisms that could differ in frequency between the ethnicities. One of the largest eQTL studies of peripheral blood samples was recently performed on Dutch and UK populations and controlled for probe biases. They, identified eQTL in six of the genes that were that were differentially expressed in our study (*SERPING1*, *DDX24*, *OAS3*, *MX1*, *ZDHHC18*, and *NUP214*; Fehrmann et al., 2011), but of these only *SERPING1* shows a significant eQTL in our Atlanta study. While we cannot rule out *cis*-eQTL contributing to divergence between the ethnic groups, they are not pervasive in the studied genes, and could not account for the covariance among genes. Individual *trans*-eQTL effects are not large or pleiotropic enough to cause the observed divergence between Melanesians and Indians, though in aggregate undetected *trans*-effects presumably account for the ethnic differences.

In both Morocco and Fiji, age and gender also have only a minor influence on the blood transcriptome. Several studies have previously (Eady et al., 2005; Karlovich et al., 2009; Xu et al., 2011; Kent et al., 2012) reported minor age-related differential expression of inflammatory genes in large population samples, and age does not appear to be a major factor in our analyses of the targeted 96 transcripts either. In our experience (Idaghdour et al., 2010, unpublished data) the variance explained is considerably smaller than lifestyle and ethic differences, and may in any case be due to correlation with metabolic parameters that differ between age classes. Our study also has a relatively small variance in age with a median of 36 years (range 21–65), so differential expression associated with immunosenescence would not be detected.

The absence of differentiation of rural and urban Melanesian Fijians is surprising given the differences in their lifestyles, the former continuing to be quite traditional with minimal influence of modern conveniences and for the most part a diet that is not based on processed and convenient foods. On the other hand, doctors working with the villagers verbally reported increasing incidence of hypertension and diabetes, metabolic diseases that were absent from the population two generations ago. This suggests that modernity is starting to impact disease profiles and this may be reflected in the Suva-like gene expression profiles. By contrast, the Indian villagers have retained their mostly vegetarian diet, use coconut oil instead of vegetable oil in their cooking, and do not show the epidemiological transition to Western disease (Jamison and Mosley, 1991; Murray and Lopez, 1997).

The rural Indians have significantly lower body mass indexes, and this likely contributes to the significant association of Axis 2 with the rural Indian versus urban contrast. It is not yet clear what molecular or cellular function Axis 2 represents. A small number of defining genes are annotated to red blood cell function and heme binding, suggesting that it represents erythrocyte function. Our previous studies (Preininger et al., submitted) have not detected any correlation between Axis 2 score and erythrocyte (or platelet) numbers, and genes that are known to be enriched in these cell types do not predict the Axis 2 score, so it is not simply a molecular reporter of red blood cell number. It seems less likely that genetic variation producing this Axis causes variation in BMI, than that elevated body fat influences gene expression in the axis. Nevertheless, people with similar body weights also differ with respect to their position along the axis, as can be seen in Figure 6, and future studies will investigate whether and how this influences the onset of metabolic syndrome diseases. An alternative possibility is that Melanesians may have evolved genetic divergence with respect to regulation of Axis 2 as a result of historical body weight patterns related to fertility-associated fattening rituals (Pollock, 1995), though this is unlikely since the high levels of obesity present on the islands are thought to be a post-World War II phenomenon (Hodge et al., 1992).

The other axes that distinguish rural Indians, Axes 5 and 6, appear to be related to inflammation/innate immune responsiveness and development of immunity respectively. Axis 5 is for example heavily enriched for genes annotated to TLR signaling. A similar study (Freedman et al., 2010) using Fluidigm to measure transcript abundance in 1,846 Framingham study participants showed inflammatory transcripts, notably TLR receptors and the NFκB pathway, to be associated with obesity and BMI, particularly when derived from platelets rather than leukocytes. Our studies seem to separate the BMI and inflammation components into different axes, and we note that platelets are mostly excluded by LeukoLOCK filters, so BMI and inflammation may subject to different lifestyle influences that are nevertheless not entirely independent owing to the multivariate nature of gene regulation. Although there is no clear gene ontology enrichment for Axis 6, re-examination of a vaccine response study (Nakaya et al., 2011) showed a strong association with production of high or low titers of influenza antibodies (M. Preininger, Artika Praveeta Nath, and Greg Gibson, in prep). Axis 6 was also observed to differentiate the rural Berbers from urban Moroccans (Idaghdour et al., 2010; Preininger et al., submitted), and notably the sign of the divergence of the rural samples is the same in both countries: in (Preininger et al., submitted) rural life was mildly positively correlated with Axis 2 and here it is negatively correlated with it, as expected given the inversion of the Axes due to the array platforms. This difference raises the possibility that there may be something of a global signature of rural versus urban living, though it seems likely that local factors are also impinging. Axis 7 is heavily enriched for interferon and anti-viral response genes, and interestingly it shows some evidence for being more strongly induced in the rural Melanesians (lower Ct values implies higher expression). This is counter-intuitive to the naïve expectation that they may be relatively unexposed to the menagerie of infections that would be expected in an urban population. In studies of infectious disease cohorts (Chaussabel et al., 2008), including Tuberculosis, Staphylococcus, and HIV, Axes 5, 6, and 7 are typically associated with active infection (in preparation). In conclusion, as in Morocco, we find a strong association between lifestyle and gene expression in peripheral blood where differences between ethnicities are only apparent in the rural populations. This conclusion should be tempered by concerns over the small sample size and RNA quality, but it is consistent with the notion that urban living pushes gene expression along common axes of variation toward a distinctive state that may represent an environmental response to modernity. Much of this likely reflects altered immune function, but the replicated association of at least one axis with a major indicator of chronic metabolic disease, hints that there is a coordinated cellular response to high body fat that may influence people’s long-term wellness and contribute to the rising morbidity known as metabolic syndrome.

## Conflict of Interest Statement

The authors declare that the research was conducted in the absence of any commercial or financial relationships that could be construed as a potential conflict of interest.

## Supplementary Material

The Supplementary Material for this article can be found online at http://www.frontiersin.org/Applied_Genetic_Epidemiology/10.3389/fgene.2012.00243/abstract

Supplementary Table S1 Raw and transformed illumina and fluidigm data.

Supplementary Table S2 Experimental design files.

Supplementary Table S3 List of probes used to measure axes of variation.

## References

[B1] Berry M. P. Graham C. M. McNab F. W. Xu Z. Bloch S. A. Oni T. (2010). An interferon-inducible neutrophil-driven blood transcriptional signature in human tuberculosis. Nature 466, 973–977 10.1038/nature09247 20725040 PMC3492754

[B2] Brian G. Ramke J. Maher L. Page A. Szetu J. (2010). The prevalence of diabetes among adults aged 40 years and over in Fiji. N. Z. Med. J. 17, 68–75 21358785

[B3] Chaussabel D. Quinn C. Shen J. Patel P. Glaser C. Baldwin N. (2008). A modular analysis framework for blood genomics studies: application to systemic lupus erythematosus. Immunity 29, 150–164 10.1016/j.immuni.2008.05.012 18631455 PMC2727981

[B4] Cheung V. G. Spielman R. S. Ewens K. G. Weber T. M. Morley M. (2005). Mapping determinants of human gene expression by regional and genome-wide association. Nature 437, 1365–1369 10.1038/nature04244 16251966 PMC3005311

[B5] Dakeshita S. Kawai T. Uemura H. Hiyoshi M. Oguma E. Horiguchi H. (2009). Gene expression signatures in peripheral blood cells from Japanese women exposed to environmental cadmium. Toxicology 257, 25–32 10.1016/j.tox.2008.12.004 19118595

[B6] Eady J. J. Wortley G. M. Wormstone Y. M. Hughes J. C. Astley S. B. Foxall R. J. (2005). Variation in gene expression profiles of peripheral blood mononuclear cells from healthy volunteers. Physiol. Genomics 11, 402–411 16014386 10.1152/physiolgenomics.00080.2005

[B7] Emilsson V. Thorleifsson G. Zhang B. Leonardson A. S. Zink F. Zhu J. (2008). Genetics of gene expression and its effect on disease. Nature 452, 423–428 10.1038/nature06758 18344981

[B8] Feezor R. J. Baker H. V. Mindrinos M. Hayden D. Tannahill C. L. Brownstein B. H. (2004). Whole blood and leukocyte RNA isolation for gene expression analyses. Physiol. Genomics 19, 247–254 10.1152/physiolgenomics.00020.2004 15548831

[B9] Fehrmann R. S. N. Jansen R. C. Veldink J. H. Westra H.-J. Arends D. Bonder M. J. (2011). Trans-eQTLs reveal that independent genetic variants associated with a complex phenotype converge on intermediate genes, with a major role for the HLA. PLoS Genet. 7, e1002197 10.1371/journal.pgen.1002197 21829388 PMC3150446

[B10] Fink J. Gu F. Ling L. Tolfvenstam T. Olfat F. Chin K. C. (2007). Host gene expression profiling of dengue virus infection in cell lines and patients. PLoS Negl. Trop. Dis. 1, e86 10.1371/journal.pntd.0000086 18060089 PMC2100376

[B11] Freedman J. E. Larson M. G. Tanriverdi K. O’Donnell C. J. Morin K. Hakanson A. S. (2010). Relation of platelet and leukocyteinflammatorytranscripts to body mass index in the Framingham heart study. Circulation 13, 119–129 20606121 10.1161/CIRCULATIONAHA.109.928192 PMC2910759

[B12] Fry R. C. Navasumrit P. Valiathan C. Svensson J. P. Hogan B. J. Luo M. (2007). Activation of inflammation/NF-kappaB signaling in infants born to arsenic-exposed mothers. PLoS Genet. 3, e207 10.1371/journal.pgen.0030207 18039032 PMC2082467

[B13] Gibson G. (2008). The environmental contribution to gene expression profiles. Nat. Rev. Genet. 9, 575–581 10.1038/ni0608-575 18574472

[B14] Gilad Y. Pritchard J. K. Thornton K. (2009). Characterizing natural variation using next-generation sequencing technologies. Trends Genet. 25, 463–471 10.1016/j.tig.2009.09.003 19801172 PMC3994700

[B15] Hodge A. M. Dowse G. K. Zimmet P. Z. (1992). Obesity in Pacific populations. Pac. Health Dialog. 3, 77–86

[B16] Hughes R. G. Marks G. C. (2009). Against the tide of change: diet and health in the Pacific islands. J. Am. Diet. Assoc. 109, 1700–1703 10.1016/j.jada.2009.07.015 19782168

[B17] Idaghdour Y. Czika W. Shianna K. V. Lee S. H. Visscher P. M. Martin H. C. (2010). Geographical genomics of human leukocyte gene expression variation in southern Morocco. Nat. Genet. 42, 62–67 10.1038/ng.495 19966804 PMC2798927

[B18] Idaghdour Y. Storey J. D. Jadallah S. J. Gibson G. (2008). A genome-wide gene expression signature of environmental geography in leukocytes of Moroccan Amazighs. PLoS Genet. 4, e1000052 10.1371/journal.pgen.100005 18404217 PMC2290968

[B19] Inouye M. Silander K. Hamalainen E. Salomaa V. Harald K. Jousilahti P. (2010). An immune response network associated with blood lipid levels. PLoS Genet. 6, e1001113 10.1371/journal.pgen.1001113 20844574 PMC2936545

[B20] Jamison D. T. Mosley W. H. (1991). Disease control priorities in developing countries: health policy responses to epidemiological change. Am. J. Public Health 81, 15–22 10.2105/AJPH.81.1.15 1983911 PMC1404931

[B21] Karlovich C. Duchateau-Nguyen G. Johnson A. McLoughlin P. Navarro M. Fleurbaey C. (2009). A longitudinal study of gene expression in healthy individuals. BMC Med. Genomics 2, 33 10.1186/1755-8794-2-33 19500411 PMC2713969

[B22] Kawai T. Morita K. Masuda K. Nishida K. Shikishima M. Ohta M. (2007). Gene expression signature in peripheral blood cells from medical students exposed to chronic psychological stress. Biol. Psychol. 76, 147–155 10.1016/j.biopsycho.2007.07.008 17766027

[B23] Kent J. W. Jr. Göring H. H. Charlesworth J. C. Drigalenko E. Diego V. P. Curran J. E. (2012). Genotype × age interaction in human transcriptional ageing. Mech. Ageing Dev. 133, 581–590 10.1016/j.mad.2012.07.005 22871458 PMC3541784

[B24] Kim J. Gibson G. (2010). Insights from GWAS into the quantitative genetics of transcription in humans. Genet. Res. 92, 361–369 10.1017/S001667231000056X 21429268

[B25] Lako V. (2001). Dietary trend and diabetes: its association among indigenous Fijians 1952 to 1994. Asia. Pac. J. Clin. Nutr. 10, 183–187 10.1046/j.1440-6047.2001.00255.x 11708305

[B26] Lal B. V. (1986). Murmurs of dissent: Non-resistance on Fiji plantations. Hawaiian J. Hist. 20, 188–214

[B27] Mason E. Tronc G. Nones K. Matigian N. Kim J. Aranow B. J. (2010). Transmission of leukocyte gene expression profiles in population samples from Brisbane, Australia. PLoS ONE 5, e14479 10.1371/journal.pone.0014479 21217831 PMC3013110

[B28] Mavoa H. M. McCabe M. (2008). Sociocultural factors relating to Tongan’s and Indigenous Fijians’ pattern of eating, physical activity and body size. Asia Pac. J. Clin. Nutr. 17, 375–384 18818156

[B29] Monks S. A. Leonardson A. Zhu H. Cundiff P. Pietrusiak P. Edwards S. (2004). Genetic inheritance of gene expression in human cell lines. Am. J. Hum. Genet. 75, 1094–1105 10.1086/426461 15514893 PMC1182144

[B30] Murray C. J. Lopez A. D. (1997). Mortality by cause for eight regions of the world: global burden of disease study. Lancet 349, 1269–1276 10.1016/S0140-6736(05)62208-8 9142060

[B31] Myers A. J. Gibbs J. R. Webster J. A. Rohrer K. Zhao A. Marlowe L. (2007). A survey of genetic human cortical gene expression. Nat. Genet. 39, 1494–1499 10.1038/ng.2007.16 17982457

[B32] Nakaya H. I. Wrammert J. Lee E. K. Racioppi L. Marie-Kunze S. Haining W. N. (2011). Systems biology of vaccination for seasonal influenza in humans. Nat. Immunol. 12, 786–795 10.1038/ni.2067 21743478 PMC3140559

[B33] Peretz A. Peck E. C. Bammler T. K. Beyer R. P. Sullivan J. H. Trenga C. A. (2007). Diesel exhaust inhalation and assessment of peripheral blood mononuclear cell gene transcription effects: an exploratory study of healthy human volunteers. Inhal. Toxicol. 19, 1107–1119 10.1080/08958370701665384 17987463

[B34] Pollock N. J. (1995). Cultural elaborations of obesity – fattening practices in Pacific societies. Asia Pac. J. Clin. Nutr. 4, 357–360 24394425

[B35] Powell J. E. Henders A. K. McRae A. F. Wright M. J. Martin N. G. Dermitzakis E. T. (2012). Genetic control of gene expression in whole blood and lymphoblastoid cell lines is largely independent. Genome Res. 22, 456–466 10.1101/gr.126540.111 22183966 PMC3290781

[B36] Qin S. P. Kim J. Arafat D. Gibson G. (2012). Effect of normalization on statistical and biological interpretation of gene expression profiles. Front. Genet. 3, 160 10.3389/fgene.2012.00160 PMC3668151 23755061

[B37] Saito S. (1995). 1993 National Nutrition Survey. Suva: National Food and Nutrition Centre

[B38] Schadt E. E. Molony C. Chudin E. Hao K. Yang X. Lum P. Y. (2008). Mapping the genetic architecture of gene expression in human liver. PLoS Biol. 6, e107 10.1371/journal.pbio.0060107 18462017 PMC2365981

[B39] Schultz J. T. Vatucawaqa P. Tuivaga J. (2007). 2004 Fiji National Nutrition Survey. Suva: National Food and Nutrition Centre

[B40] Siefkenm K. Macniven R. Schofield G. Bauman A. Waqanivalu T. (2012). Stocktake of physical activity programs in the Pacific Islands. Health Promot. Int. 27, 197–207 10.1093/heapro/dar026 21561985

[B41] Skelly D. A. Ronald J. Akey J. M. (2009). Inherited variation in gene expression. Annu. Rev. Genomics Hum. Genet. 10, 313–332 10.1146/annurev-genom-082908-150121 19630563

[B42] Spurgeon S. L. Jones R. C. Ramakrishnan R. (2008). High throughput gene expression measurement with real time PCR in a microfluidic dynamic array. PLoS ONE 3, e1662 10.1371/journal.pone.0001662 18301740 PMC2244704

[B43] Thaman R. R. (1988). Consumerism, the media and malnutrition in the Pacific Islands. J. Pacific Stud. 14, 68–90

[B44] Ulijaszek S. (2009). Modernization, migration and nutritional health of Pacific Island populations. Environ. Sci. 12, 167–176 16077468

[B45] Xu Q. Ni S. Wu F. Liu F. Ye X. Mougin B. (2011). Investigation of variation in gene expression profiling of human blood by extended principle component analysis. PLoS ONE 6, e26905 10.1371/journal. pone.0026905 22046403 PMC3203156

